# The Matron Abroad

**Published:** 1907-09-14

**Authors:** 


### Nursing Administration.

### THE MATRON ABROAD.

When people begin to catalogue the qualities indispensable to the nurse, it is long before they come to an end. The good nurse, like the good woman, ought indeed to be perfect, and it would be hard to name any defect we should be content to find in her. And yet when it comes to choosing a nurse to fill an appointment abroad, it becomes clear that over and above the qualities of the good nurse there must be something higher still. The difficulties of the matron who is placed in a position of authority in a strange land are baffling because they cannot be foreseen. It is impossible to prepare beforehand for conditions of living which will in all probability turn out different to anything that has been imagined. Just as the casual visitor to India invariably finds that she has chosen all her clothes amiss, however much advice she may have sought on the subject, so the matron called to duty abroad is certain to find she has overlooked contingencies she would give much to have provided against, and that many of her anxious preparations have been wide of the mark. It follows, then, that the matron abroad must be a woman of resource. The routine people who expect things to go smoothly because they have decreed that such shall be the case, are of little use in countries where routine has not yet been born, and where the day's difficulties have to be met on their own merits, every day fresh. But although the gift of resource is golden, it is even less essential to the matron abroad than the steadiness of character which is a law to itself, and which will in the long run outwear all the more brilliant qualities of clever people. Resource can be gained by hard experience. But it is very rarely indeed that nature puts ballast into heads which have started life without it. Whatever may be the case with the perfect nurse, it would seem that for the perfect matron set over a hospital or nursing institute abroad it is a sense of administrative responsibility which is the one thing to be sought for and patiently developed.

After the first novelty has worn off, the most striking feature of hospital work in partly settled countries is the enormous pressure of mechanical detail which falls to the lot of the head. All the petty things which get done at home as a matter of course are recurrent problems in a place where the white population is an inconsiderable fragment perhaps among coloured races. Hence the day's duties are often made up of trivial work such as would be the lot of the wardmaids, porters, or probationers at home, while the really important things have to be fitted in when spare moments can be found. Often it may happen that the matron will find no previous attempt at organisation; only a general scramble to get things done. There may be no rules, no books, no system of any kind. And under a pressure of perpetual emergencies the work of organisation has to be put in hand, and a foundation laid upon which another may in time to come find it possible to build. Where the climate is bad and the matron is but a bird of passage, it is all the more important that her stay should tell for good, that she should utilise her predecessor's work as far as possible, and that she should leave matters so that her successor shall not need to begin fresh all over again. To work as part of a general scheme is better policy in a short appointment than to inaugurate novelties which cannot be maintained without exceptional talents.

The great problem which besets the superintendent in foreign countries is that of dealing with the untrained material. There are places where helpers who can acquire the elements of hospital work are hard to get, and where the simplest nursing duties can be entrusted solely to Europeans already tained. Yet even with the most unpromising material perseverance will effect much. The matron's success will depend in considerable degree on her talent for acquiring the language around her, as this can alone give access to the ideas which lie behind the apparent denseness of the unknown race. Certainly wonderful progress has been made of late years in the most unlikely quarters in training natives to act as valuable assistants to trained nurses. Much more might be done if the dictum were once abjured that such and such people can never be taught to be nurses. It is true that the veneer of civilisation is very thin among some races who have been educated to familiarity with all the latest developments of science. The squaw in her blanket may be found taking kodak portraits, and the judge may dine one night with fashionably attired ladies who may next morning appear, bleeding and dishevelled before him in court, charged with brawling in the streets. The matron who aspires to take her part in the great work of advancing the cause of true civilisation in distant lands will need all her patience, all her tact, all the boundless resources which education possesses over the untutored mind, if she is to introduce the art of nursing among such people, unaccustomed to the restraint which it demands. But should she be even partially successful she will have done more for the race than generations of untrained enthusiasts have been able to effect. Every woman who has been able to acquire the simplest principles of cleanliness under the eye of a trained nurse leaves the hospital to be a centre of enlightenment to a wide circle who might possibly never come under direct European influence. To be for ever hopeful is to pave the way for understanding something of the common humanity which lies in unexpected places, ready to yield a rich reward. How clearly some people perceive the radical deficiencies of native races. How incisively the typical well-trained Englishwoman will tell you of "their" traits, the reasons why "they" can never be trusted. And how striking the contrast between the sweet cordiality of Miss Colenso's "We Zulus," or the racy humour of Miss Kingsley ever ready to turn the laugh against herself in her struggles to sound the depths of a race she had learned to love.

